# Suppression of type 1 pilus assembly in uropathogenic *Escherichia coli* by chemical inhibition of subunit polymerization

**DOI:** 10.1093/jac/dkt467

**Published:** 2013-12-08

**Authors:** Alvin W. H. Lo, Karen Van de Water, Paul J. Gane, A.W. Edith Chan, David Steadman, Kiri Stevens, David L. Selwood, Gabriel Waksman, Han Remaut

**Affiliations:** 1 Structural and Molecular Microbiology, VIB Department of Structural Biology, VIB, Pleinlaan 2, 1050 Brussels, Belgium; 2 Structural Biology Brussels, Vrije Universiteit Brussel, Pleinlaan 2, 1050 Brussels, Belgium; 3 Wolfson Institute for Biomedical Research (WIBR), UCL, Gower Street, London WC1E 6BT, UK; 4 Institute of Structural and Molecular Biology (ISMB), UCL/Birkbeck College, Malet Street, London WC1E 7HX, UK

**Keywords:** chaperone–usher pathway, urinary tract infections, type 1 pili, UPEC, anti-virulence

## Abstract

**Objectives:**

To identify and to characterize small-molecule inhibitors that target the subunit polymerization of the type 1 pilus assembly in uropathogenic *Escherichia coli* (UPEC).

**Methods:**

Using an SDS–PAGE-based assay, *in silico* pre-filtered small-molecule compounds were screened for specific inhibitory activity against the critical subunit polymerization step of the chaperone–usher pathway during pilus biogenesis. The biological activity of one of the compounds was validated in assays monitoring UPEC type 1 pilus biogenesis, type 1 pilus-dependent biofilm formation and adherence to human bladder epithelial cells. The time dependence of the *in vivo* inhibitory activity and the overall effect of the compound on UPEC growth were determined.

**Results:**

*N*-(4-chloro-phenyl)-2-{5-[4-(pyrrolidine-1-sulfonyl)-phenyl]-[1,3,4]oxadiazol-2-yl sulfanyl}-acetamide (AL1) inhibited *in vitro* pilus subunit polymerization. In bacterial cultures, AL1 disrupted UPEC type 1 pilus biogenesis and pilus-dependent biofilm formation, and resulted in the reduction of bacterial adherence to human bladder epithelial cells, without affecting bacterial cell growth. Bacterial exposure to the inhibitor led to an almost instantaneous loss of type 1 pili.

**Conclusions:**

We have identified and characterized a small molecule that interferes with the assembly of type 1 pili. The molecule targets the polymerization step during the subunit incorporation cycle of the chaperone–usher pathway. Our discovery provides new insight into the design and development of novel anti-virulence therapies targeting key virulence factors of bacterial pathogens.

## Introduction

For many pathogenic bacteria, the contact interface with their hosts is formed by proteinaceous hair-like cell surface appendages termed pili or fimbriae. In Gram-negative bacteria, these structures are assembled through a number of dedicated secretion pathways that play a crucial role in mediating bacterial traits associated with virulence, including host cell adherence and/or invasion, biofilm formation and transportation of DNA or proteins across membranes.^1^ The prospect of disrupting the biogenesis or function of these nanomachineries has garnered increasing interest as an anti-virulence approach against pathogenic bacteria. The growing knowledge of the structural basis and molecular processes steering the assembly and secretion of these virulence structures and their prevalence in clinically relevant pathogens render them a promising target for development of novel therapeutic agents.^2–4^

Uropathogenic *Escherichia coli* (UPEC) is the major aetiological agent of urinary tract infections (UTIs) and it is estimated to affect 150 million individuals globally per annum.^5^ The use of available antibiotics has led to significant improvements in the management of UTIs; however, recurrent infections^6^ and an increasing resistance to conventional antibiotics, as exemplified by the recent pandemic of the multidrug-resistant UPEC strain ST131,^7–9^ are a cause of major concern. UPEC also form a burden in hospital or nursery wards, representing up to 30% of nosocomial infections, especially in patients with urinary catheters.^10^ The indispensable steps in the onset and persistence of UPEC infections are the attachment and invasion of bladder epithelial cells and the establishment of biofilm-like intracellular bacterial communities.^11–13^ These steps are crucially dependent on the presence of type 1 pili and previous efforts to impair their assembly or adhesive function have yielded several promising antagonists (reviewed in Lo *et al*.,^4^ Cegelski *et al*.^14^ and Waksman and Hultgren^15^). Here, we describe the identification and characterization of a small-molecule inhibitor that impedes the biogenesis of type 1 pili by targeting subunit polymerization in an early assembly step.

**Figure 1.**
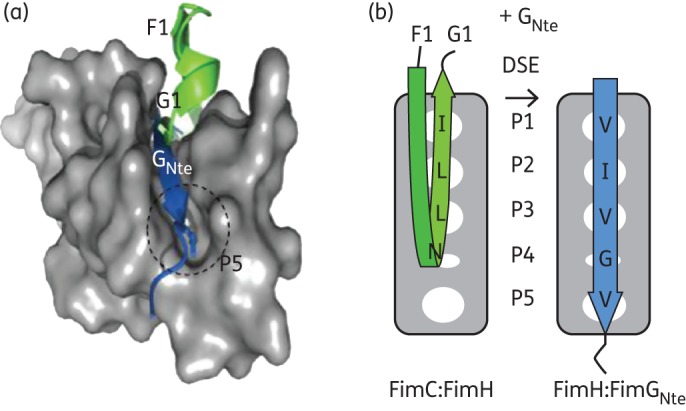
Identification of pilus polymerization inhibitors. Structure (a) and schematic representation (b) of the FimH pilin domain (shown as grey molecular surface, encompassing residues 158 to 279 of PDB:1ZE3) in complex with the FimC F1–G1 strands (green) or FimG Nte peptide (blue; taken from PDB:3JWN). The P5 pocket is labelled and the P5 binding residue (Val_11_) is shown as sticks. (b) P1 to P5 pockets are shown as white ovals and the interacting residues in the FimC F1 strand or FimG_Nte_ are labelled.

Type 1 pili are assembled by a highly conserved biosynthetic assembly mechanism termed the chaperone–usher pathway. Chaperone–usher pathways are responsible for the biogenesis of a diverse arsenal of virulence-associated cell surface organelles present in a myriad of pathogenic γ-proteobacteria.^1,16^ Besides the adhesin and structural pilus subunits, they comprise two accessory components: a periplasmic chaperone captures nascent subunits as they emerge from the inner membrane translocon and shuttles them to an outer membrane assembly platform, the usher, where pilus subunits are incorporated into the base of the growing fibre and subsequently translocate across the outer membrane.^17–19^ Chaperone–usher pilus subunits are characterized by an incomplete immunoglobulin (Ig) fold lacking the C-terminal β-strand, and by the presence of a disordered extension of 10–20 residues at the N-terminus.^20,21^ In the periplasm, pilus subunits are stabilized by the chaperone, which donates an extended β strand (strand G1) to complement the missing structural information in the subunit Ig fold.^20,21^ At the usher, subunits undergo non-covalent polymerization through a similar fold complementation mechanism, now involving the N-terminal extension peptide (Nte) of the newly incoming pilus subunit (Figure S1, available as Supplementary data at *JAC* Online). These Nte sequences contain a conserved motif of alternating hydrophobic residues termed ‘P2–P5 residues’ that make knobs into hole-packing interactions with the equivalent hydrophobic pockets in the acceptor groove of the pilus subunit (Figure 1a and b). In the chaperone–subunit interaction, the G1 strand occupies pockets P1–P4 and leaves P5 accessible to the solvent (Figure 1a and b). During subunit polymerization, the chaperone G1 donor strand bound to the subunit at the base of the pilus is exchanged for the Nte of the newly recruited chaperone:subunit complex, a process called ‘donor strand exchange’ (DSE).^22^ DSE occurs in a concerted ‘zip-in zip-out’ mechanism that involves the formation of a transient ternary complex between the chaperone:subunit complex and the incoming Nte.^23^ DSE ternary complex formation is initiated by the docking of the Nte P5 residue to the P5 pocket on the acceptor chaperone:subunit complex.^23^

The adhesive subunit, FimH, constitutes the first subunit to be incorporated, is present in a single copy and is crucial for the activation of the FimD usher for pilus assembly.^24^ In addition, genetic inactivation of FimG and/or FimF, the subunits succeeding FimH and forming the link between the adhesin and the FimA pilus shaft, leads to polymerization arrest and the accumulation of FimD:FimC:FimH complexes unable to promote mannose-sensitive haemagglutination.^25^ Hence, we speculated that the chemical inhibition of the DSE reaction between FimH and FimG would prevent FimG incorporation into the pilus as well as that of downstream subunits. We reasoned that chemical compounds that are able to competitively interact with the P5 pocket would serve as pilus polymerization inhibitors.

Here, we performed structure-based *in silico* screens of chemical libraries to derive a filtered set of compounds with predicted complementarity to the FimC:FimH P5 pocket area, which were subsequently tested for *in vitro* DSE inhibition. Using this approach, we have identified a compound that is able to inhibit the DSE reaction between FimH and FimG Nte in a concentration-dependent manner. Bacteria exposed to the inhibitor were found to be devoid of type 1 pili or surface-exposed FimH. We further show that the inhibitor impedes the type 1 pilus-dependent virulence traits critical for UPEC pathogenesis, including biofilm formation and adherence to human bladder epithelial cells. Strikingly, the disruption of UPEC type 1 pili appears to occur almost instantaneously upon addition of the compound, suggesting that the activity may not be restricted to *de novo* synthesis, but extends to pre-formed pili. To the best of our knowledge, this is the first report of a small-compound molecule that specifically targets the polymerization step in the pilus subunit incorporation cycle. Collectively, this study provides the basis for the design and development of compounds for therapeutic intervention against other clinically significant pathogens.

## Materials and methods

### Bacterial strains

Uropathogenic *E. coli* UTI89, UTI89 Δ*fimA*–*H* and *E. coli* BL21 (DE3) are described in Table S1 (available as Supplementary data at *JAC* Online).

### Construction, expression and purification of FimC_his_:FimH

To facilitate the cloning of *fimH* and *fimC* for co-expression, the MultiSite Gateway^®^ Three-Fragment Vector Construction Kit (Invitrogen) was used. The full coding sequence of *fimC* with a C-terminal His tag was amplified from pETS1000^26^ using primers FimCf and FimCr (Table S1, available as Supplementary data at *JAC* Online) and cloned into pDONRP4-P1R donor vector via *attB/attP* recombination, resulting in pKVWc1. The full coding sequence of *fimH* was amplified using FimH3 and FimH7 primers and cloned into pDONR221 via BP recombination, yielding pENT53. To monitor the protein complex yield, the green fluorescent protein gene *gfp*+^27^ was amplified from pWH1012gfp+ using gfp7 and gfp8 primers and cloned into pDONRP2R-P3, yielding pENT105. Subsequently, pKVWc1, pENT53 and pENT105 underwent *attL/attR* recombination in the presence of pDEST22a to generate pKVWe1 expression vector. The pDEST22a was derived from pET22b (Novagen) digested with XbaI and EcoRI and followed by a fill-in reaction to generate blunt ends. The attR4-ccdB-CmI-attR3 cassette was amplified from pDEST R4–R3 using primers FP24 and RP24 and ligated into the linearized pET22b, resulting in pDEST22a.

*E. coli* BL21 (DE3) harbouring pKVWe1 was grown at 37°C in M9 minimal medium supplemented with 0.4% glucose, 20 mM MgCl_2_, 100 μg/mL ampicillin and 1% (v/v) vitamin solution. Cells were induced at an OD_600_ of 1 with 1 mM IPTG at 37°C (where OD stands for optical density). Periplasmic extraction of cells incubated overnight was carried out. The FimC_his_:FimH complex was purified using a Ni-NTA column (Qiagen). The complex was eluted with 200 mM imidazole in 20 mM Tris–HCl, pH 8.8/250 mM NaCl buffer. Fractions containing the FimC_his_:FimH complex as judged by SDS–PAGE were pooled and dialysed against 20 mM MES, pH 6/10 mM NaCl overnight at 4°C. The final polishing step was done using a HiTrap SP FF column (GE Healthcare) at room temperature and subjected to 10 mM–1 M NaCl gradient elution. FimC_his_:FimH was eluted at 400 mM NaCl and dialysed against 20 mM Tris, pH 8.8/10 mM NaCl overnight at 4°C.

### Virtual screening

Two docking strategies were employed. First, the docking area was limited to the P5 pocket as found in the chaperone:subunit complex in PDB:1ze3 (Figure S2a, available as Supplementary data at *JAC* Online). Second, compounds were allowed to dock in the more extended donor strand acceptor groove (Figure S2b, available as Supplementary data at *JAC* Online), representing a binding mode in which they induce full or local displacement of the chaperone donor strand. In practice, FimC (chain C in PDB:1ze3) was removed from the crystal structure to create the extended groove. FimC was omitted from the complex and a hydrophobic anchor was placed at the P5 site to allow for docking to the full binding groove extending into P3 and P4 pockets (Figure S2b, available as Supplementary data at *JAC* Online). The anchor was used to force binding near the P5 site.

A set of 72 000 small molecules was assembled from an Asinex diverse library^28^ (30 000 compounds) and a Chembridge CNS/Novacore/Diverset structurally diverse library (42 000 compounds) and was utilized for the *in silico* screen. These compounds are compliant with Lipinski's rule-of-five criteria and considered drug-like.^28,29^ Docking was performed in two stages. FRED was used for the whole set of the data as this is a very fast program. Ten energy-minimized conformers for each chemical compound were generated using MOE^30^ as initial structures for FRED.^31^ Chemscore^32^ was used as scoring function in FRED. The best 5000 hits from FRED were docked again using GOLD.^33^ GOLD generates multiple low-energy conformers on the fly. The GOLD docking was performed using GOLD's genetic algorithm (GA) with scoring function GoldScore.^34^

### In vitro DSE inhibition assay

**Figure 2.**
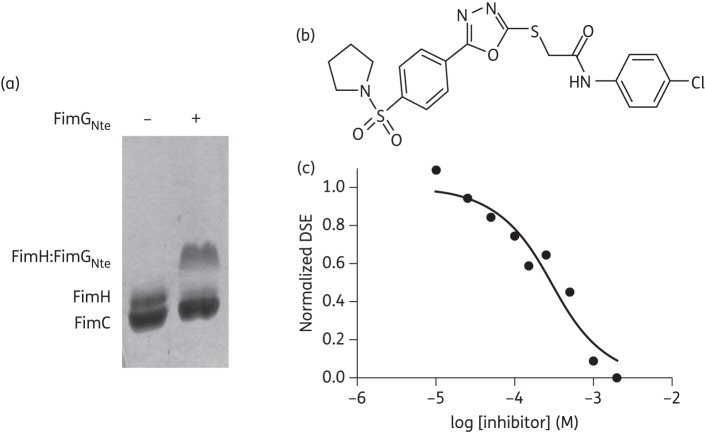
Inhibition of *in vitro* DSE. (a) SDS–PAGE showing FimC:FimH DSE with FimG_Nte_ after a 12 h incubation. FimH:FimG_Nte_ forms an SDS-stable complex that shows a mobility shift compared with FimH. (b) Structure of AL1. (c) Concentration–response curve for *in vitro* FimC:FimH–FimG_Nte_ DSE in the presence of AL1 (0.001–2 mM). DSE progression as a function of AL1 concentration, presented as adjusted volume of pixels of the DSE product (FimH:FimG_Nte_) over the adjusted volume of pixels of the total protein (FimC + FimH + FimH:FimG_Nte_) and normalized over non-treated DSE.

FimG:FimH DSE reactions were initiated by addition of 50 μM FimG N-terminal extension peptide (FimG_Nte_: ADVTITVNGKVVAKP-amide) to 2 μM FimC_his_:FimH complex in 100 mM Tris–HCl, pH 8.8/20 mM NaCl buffer and incubated for 12 h at 37°C. To screen for DSE inhibitors, DSE assays were performed in the presence of the pre-selected compounds at a final concentration of 500 μM each. The DSE reactions were then loaded onto SDS–PAGE. The Coomassie-stained gels were scanned using Bio-Rad Quantity One^®^ Chemi Doc XRS with automated sequential time exposures. The intensity of the bands corresponding to FimC_his_, FimH (the DSE substrate) and the SDS-resistant FimH:FimG_Nte_ complex (DSE product) (Figure 2a) was quantified using Bio-Rad Quantity One^®^ analysis software as a function of volume of pixels (intensity/mm^2^). The total intensity within a defined border around the bands without overlapping nearby bands was measured using the Volume Rectangle Tool. DSE completion was calculated by the following equation: adjusted volume of pixels (FimH:FimG_Nte_)/adjusted volume of pixels (FimC + FimH + FimH:FimG_Nte_). The screening hit *N*-(4-chloro-phenyl)-2-{5-[4-(pyrrolidine-1-sulfonyl)-phenyl]-[1,3,4]oxadiazol-2-yl sulfanyl}-acetamide (AL1) was re-ordered from Asinex Corporation (ASN 03798371) at ≥97% purity (HPLC) and synthesized in house (see additional methods, available as Supplementary data at *JAC* Online) in order to confirm the chemical identity of the biologically active compound.

Further DSE assays were performed in the presence of the hit compound at concentrations ranging from 0.001 to 2 mM in 20% (v/v) DMSO to determine the IC_50_ values. DSE completion for compound-treated samples was normalized over that of the non-treated control and plotted as a function of compound concentration. The IC_50_ values were calculated by non-linear regression analysis using GraphPad Prism. DMSO (20% v/v) was the negative control.

### Biofilm formation assay

Static biofilm formation was assayed as described by O'Toole and Kolter.^35^ Briefly, an overnight culture of UPEC was diluted 1/1000 with fresh Lysogeny broth (LB) supplemented with compound and incubated in a 96-well microtitre plate at room temperature for 48 h. Wells were then washed twice with PBS and stained with 0.1% (w/v) crystal violet. Following two PBS washes, biofilm-adsorbed crystal violet was extracted in 150 μL of ethanol and quantified by absorbance at 595 nm. Absorbance measurements were background corrected and are presented as the percentage of biofilm formation following normalization to non-compound-treated UTI89 or UTI89_LON^36^ cultures. The IC_50_ values and 95% CIs were calculated from the concentration–response curves by non-linear regression analysis using GraphPad Prism (a Hill slope of −1.4).

### ELISA with anti-FimH antibody

Bacterial strains were grown in LB in the presence of 50 and 200 μM AL1 at room temperature for 48 h. For FimH detection by whole-cell ELISA (WCE), Nunc MaxiSorp^TM^ flat-bottom 96-well plates (eBioscience) were coated with bacteria washed in PBS. The OD_600_ of each well was measured and plates were incubated at room temperature for 2 h. The wells were washed twice in PBS and blocked with 1% BSA for 30 min. The presence of type 1 pili was detected using a rabbit polyclonal anti-FimH antibody followed by alkaline phosphate-conjugated goat anti-rabbit IgG (Sigma). The reaction was developed in the presence of 5-bromo-4-chloro-3-indolyl-phosphate/nitro blue tetrazolium substrate (Roche) and absorbance was read at 405 nm.

### Electron microscopy visualization of bacterial piliation

Treated and non-treated samples at room temperature for 48 h were fixed with 1% (v/v) paraformaldehyde and stained with 0.5% (w/v) uranyl acetate for visualization by electron microscopy (120 kV Jeol) in order to quantify the amount of piliated versus non-piliated bacterial cells in each sample. One hundred randomly chosen bacterial cells were counted for each sample.

### Pilus dislocation assays

Overnight bacterial cultures were diluted 1000-fold with fresh LB medium and grown to an OD_600_ of 0.5 at 37°C. Bacteria were then treated with 200 μM of compound. Cultures were sampled at 1, 30 and 60 min after addition of compound or 1% DMSO for control samples. Samples were washed and suspended in PBS at an OD_600_ of 0.1. WCE was performed using anti-FimH antibody to detect the presence of type 1 pili as described above.

### Yeast agglutination assays

Yeast agglutination was assayed in 96-well U-bottom microtitre plates by mixing 35 μL of a 0.1 OD_600_ bacterial suspension sampled from cultures at 1, 30 and 60 min after addition of compound (200 μM) or 1% DMSO (control), with 10 μL of 2% (w/v) baker's yeast suspended in 20 mM sodium phosphate, pH 7.5/150 mM NaCl buffer. Following 5 min of incubation, yeast agglutination was visualized using a Moticam 2000 camera. The agglutination titre represents the maximum serial dilution of the 0.1 OD_600_ bacterial suspension that still gives yeast agglutination.

### Human urothelial cell adherence assay

The adhesion of compound-treated UTI89 to human urinary bladder epithelial cell line 5637 (ATCC HTB-9) was assessed using the protocol described by Wellens *et al.*^37^ Briefly, bacteria were grown in the presence or absence of 200 μM of compound at room temperature for 48 h. Bacteria were washed and suspended in PBS to an OD_600_ of 0.5. A total of 10^6^ to 10^7^ bacteria were added to each well of a 24-well plate (Falcon BD) containing a confluent culture of bladder cells. Plates were shaken gently at room temperature for 15 min to allow binding of the bacteria to the bladder cells. Unattached bacterial cells were removed by five washes in PBS. Bladder cell lysis and detachment from culture plates was induced by incubation with 0.25% (w/v) trypsin/2 mM EDTA for 15 min and then terminated by addition of 10% fetal calf serum. Bacterial cfu in the bladder cells lysates were determined by 10-fold serial dilutions in PBS, plated onto LB agar plates. UTI89 Δ*fimA*–*H* and UTI89 grown in the presence of 1% (v/v) DMSO were used as controls.

### Pilus fate assays

To assess the effect of AL1 on pilus quaternary structure, type 1 pili were isolated from UTI89_LON by heat extraction and MgCl_2_ precipitation as described by Brinton.^38^ Briefly, overnight bacterial cultures were diluted 1000-fold with fresh LB medium to a total of 8 L and grown to an OD_600_ of 1 at 37°C. Bacterial cells were washed twice and suspended in PBS at 4°C. Cell suspensions were heat shocked at 65°C for 30 min. The cells were pelleted and MgCl_2_ was added to the collected supernatant to a final concentration of 0.1 M and incubated for at least 1 h at 4°C to aggregate the type 1 pili. The mixture was spun down and the precipitated pili were resuspended in deionized water. Pili were treated with 200 and 500 μM AL1 for 5 min. The MgCl_2_-precipitated supernatant of heat-shocked UTI89 Δ*fim* cells was used as negative control. Boiled (5 min, 95°C) and non-boiled samples (in 1× NuPage^®^ LDS Sample Buffer) were then resolved on SDS–PAGE gels and transferred onto polyvinylidene difluoride membrane using a Mini Trans-Blot electrophoretic transfer cell (Bio-Rad Laboratories) at 100 V for 1 h. Blots were probed with rabbit polyclonal antibody against FimC:FimH complex (this study) followed by alkaline phosphatase-conjugated goat anti-rabbit IgG (Sigma).

To determine the effect of AL1 on mature type 1 pili present on the bacterial surface, a total of 300 mL of UTI89_LON culture was grown to an OD_600_ of 1 at 37°C. The cells were washed twice and suspended in 4 mL of PBS. A total of 200 mM compound was added to the cell suspension and incubated for 1 min. The cells were removed from the suspension by 20 min of centrifugation at 10 000 **g**. MgCl_2_ was added to the collected supernatant to a final concentration of 0.1 M and incubated for at least 1 h at 4°C before ultracentrifugation at 20 000 **g**. Pellets were suspended in deionized water to a volume that was scaled according to the total volume of culture grown for a direct comparison with the above type 1 pilus isolation experiment. Western blot analysis was performed as described above. Western blots were scanned using Bio-Rad Quantity One^®^ Chemi Doc XRS. The intensity of the bands was then quantified using Image Studio Lite software (LI-COR Biosciences) and expressed as sum pixel intensity values within a defined border minus background pixel intensity. The ratio of FimC to FimH for the different pilus isolates was normalized against the ratio of FimC_his_ to FimH for the purified FimC_his_: FimH complex, since the latter is known to represent FimC and FimH equimolar concentration.

### Statistical analysis

Data were expressed as the sample mean ± SEM. The difference between two samples was assessed by two-tailed unpaired Student's *t*-test, with *n* indicated for each experiment (α is taken as 0.05). Individual experiments were performed on different days, using independent starter cultures. In each experiment, multiple samples were analysed or measured as indicated.

## Results and discussion

### Structure-based in silico screening

To achieve more focused screening, chemical screening libraries were pre-filtered using structure-based *in silico* docking with the presupposition that compounds that have the potential to bind to the P5 pocket would disrupt DSE. In the structure of the usher-bound FimC:FimH complex (PDB:1ZE3),^39^ the P5 pocket forms a hydrophobic, solvent-exposed pocket with approximate dimensions of 8 Å long × 7 Å wide × 7 Å deep (Figures 1a and S2a and b, available as Supplementary data at *JAC* Online). For virtual screening, two binding hypotheses were used. First, the docking area was limited to the P5 pocket as found in the chaperone:subunit complex; second, compounds were allowed to dock in the more extended donor-strand acceptor groove, representing a binding mode in which they induced full or local displacement of the chaperone donor strand (Figure S2a and b, available as Supplementary data at *JAC* Online). For initial virtual screening, 72 000 diverse commercial compounds (see the Materials and methods section) that have rule-of-five properties were used. Ten energy-minimized conformers for each chemical compound were generated using MOE.^30^ Docking was performed with FRED^31^ and GOLD.^33^

### DSE mechanism as the target for AL1 inhibition of pilus subunit polymerization

To screen for type 1 pilus DSE inhibitors we employed an SDS–PAGE-based assay that monitored the formation of an SDS-stable complex between FimH and the FimG Nte (Figure 2a; see the Materials and methods section). The 2000 compounds that scored most favourably both in FRED and GOLD were taken through to *in vitro* screening in the FimG:FimH DSE assay at 500 μM final concentration, each being incubated for 12 h at 37°C. We identified 20 compounds that resulted in a >50% reduction in FimG:FimH complex formation under the screening conditions employed (data not shown), amounting to a hit rate of 1% in the *in silico*-filtered library. Of these, the most potent hit, AL1 (Figure 2b), was taken forward for detailed characterization. DSE reactions of FimC:FimH and FimG Nte were carried out in the presence of AL1 at various concentrations, and showed that the compound exhibited a concentration-dependent inhibition of DSE product formation, with an IC_50_ value of 286 μM (95% CI 192–425 μM; *R*^2^ = 0.94) (Figures 2c and S3, available as Supplementary data at *JAC* Online).

### Inhibition of type 1 pilus-dependent biofilm formation

**Figure 3.**
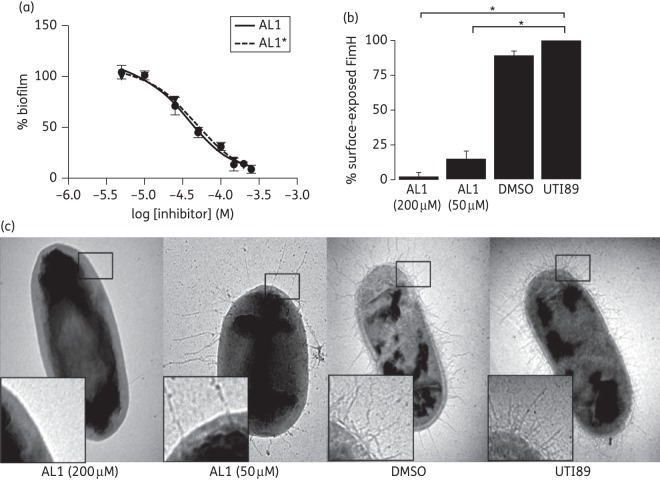
*In vivo* pilus biogenesis inhibition by AL1. (a) Inhibition of UTI89 type 1 pilus-dependent biofilm formation in the presence of AL1 and AL1* (commercial and resynthesized, respectively) at various concentrations, plotted as the percentage of biofilm formation relative to the non-treated UTI89. Data are presented as sample mean ± SEM, *n* = 3. Curves show non-linear regression fits with a Hill slope of −1.4. UTI89 Δ*fim* was deficient in biofilm formation under the same static growth condition (Figure S4a, available as Supplementary data at *JAC* Online). (b) Abundance of surface-exposed FimH on UTI89 grown in the presence of AL1 at 50 or 200 μM, as monitored by anti-FimH WCE and plotted as the percentage of non-treated UTI89 ± SEM (*n* = 2, each carried out in triplicate). Statistical analysis was performed using two-tailed unpaired Student's *t*-test to compare the percentage of surface-exposed FimH of non-treated UTI89 with that of AL1-treated UTI89 at 50 and 200 μM (*P = 0.0004 and *P* = 0.0002, respectively). (c) Representative electron micrographs of UTI89 grown in the presence of 50 or 200 μM AL1 + 1% DMSO, 1% DMSO and non-supplemented LB (labelled UTI89). Additional representative electron micrographic images for each sample are in Figure S6, available as Supplementary data at *JAC* Online.

In order to verify that the *in vitro* inhibition of FimG:FimH DSE translates into *in vivo* inhibition of pilus formation, UPEC strain UTI89, a cystitis isolate, was grown in presence of AL1 for 48 h at room temperature and type 1 pilus formation was monitored using a type 1 pilus-dependent biofilm formation assay.^40,41^ AL1 had no adverse effects on bacterial growth in rich media (Figure S4b, available as Supplementary data at *JAC* Online) and inhibited type 1 pilus-dependent biofilm formation of UTI89 in a concentration-dependent manner (Figure 3a). Under the conditions tested, AL1 inhibited UTI89 biofilm formation with an IC_50_ of 37 μM (95% CI 29–47 μM; *R*^2^ = 0.98; *n* = 3) (Figure 3a). For comparison we assessed the effect of AL1 on the biofilm formation of a UTI89 mutant strain that constitutively expresses type 1 pili (UTI89_LON^36^) under the same growth conditions. AL1 inhibited UTI89_LON biofilm formation in a concentration-dependent manner with an IC_50_ of 78 μM (95% CI 70–87 μM; *R*^2^ = 0.97; *n* = 3) (Figure S4c, available as Supplementary data at *JAC* Online).

In order to confirm that AL1 was responsible for the biological activity found in commercially available sources, AL1 was synthesized in house (see additional methods and Figure S5, available as Supplementary data at *JAC* Online). In-house-synthesized AL1 showed IC_50_ values of 46 μM (95% CI 35–61 μM; *R*^2^ = 0.98; *n* = 3) (AL1*; Figure 3a), closely matching those found for the commercially available material. Thus, AL1 is responsible for the biological activity and appears to reach its target efficiently in UTI89 periplasm. The mid-inhibitory concentrations for biofilm formation were ∼6-fold lower compared with that of the *in vitro* DSE assay. *In vivo*, FimG:FimH DSE is catalysed by the FimD usher and occurs on a sub-minute timescale.^42^ In contrast, *in vitro*, the non-catalysed DSE reaction requires a 25-fold excess of FimG Nte in order to reach completion over a time scale of 12 h. This excess of FimG Nte likely explains the higher IC_50_ values observed in the DSE assay.

### AL1 disrupts type 1 pilus formation in vivo

To confirm that the biofilm-deficient phenotype stems from the loss of type 1 pili, the presence of surface-exposed FimH and type 1 pili on UTI89 grown in the presence of AL1 for 48 h at room temperature was analysed by negative stain electron microscopy and anti-FimH WCE (Figure 3b). WCE revealed that, compared with DMSO-treated controls, UTI89 grown in the presence of 50 or 200 μM AL1 reduced surface-exposed FimH by ∼85% and 97%, respectively (*P* = 0.0003 and *P* = 0.0002, respectively, *n* = 2 independent experiments, each carried out in triplicate). Representative electron micrographs showed peritrichous piliation of bacteria in DMSO (control), whereas treatment with 50 or 200 μM AL1 resulted in non-piliated and sparsely piliated bacteria, respectively (Figures 3c and S6, available as Supplementary data at *JAC* Online). Moreover, the few pili that were present on the bacteria at 50 μM AL1 were aberrantly long (Figure 3c). A similar phenotype is observed in *fimH* mutant strains, where the accumulated periplasmic pool of FimA pilus subunits is directed to those few pilus assembly platforms in the outer membrane that non-specifically become activated for pilus assembly in the absence of FimH.^43^ This leads to non-physiologically long pili that have an increased tendency to break under shear stress.

### AL1 attenuates UPEC adherence to human bladder cells

**Figure 4.**
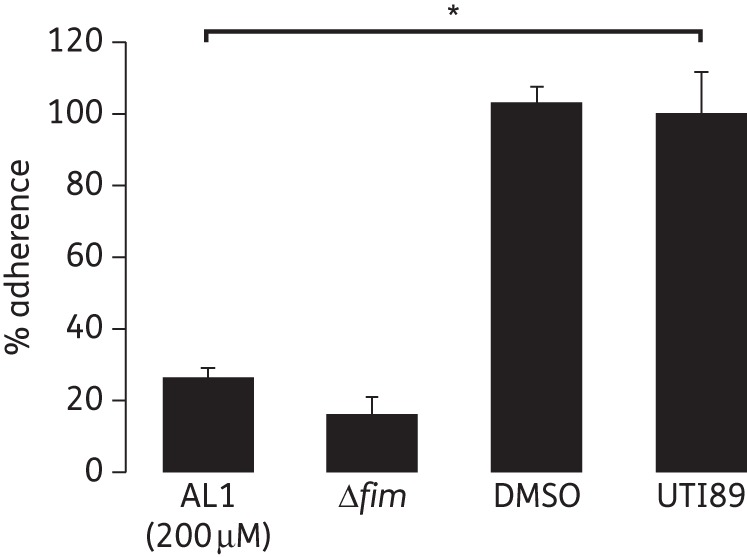
AL1 attenuates UTI89 bladder cell binding. Adherence of AL1-treated (200 μM, 1% DMSO) UTI89 and UTI89 Δ*fim* to human urinary bladder epithelial cell line 5637. Data are expressed as mean ± SEM percentage adherence relative to non-treated UTI89; *n* = 2 independent experiments with three replicates each. Statistical analyses were performed using two-tailed unpaired Student's *t*-test to compare the percentage of reduction in ability to bind to human bladder cells of AL1-treated UTI89 with that of non-treated UTI89 (*P = 0.0003).

The lack of type 1 pili renders UPEC avirulent by abrogating its ability to colonize and invade during early events of infection.^44^ UTI89 cultured in the presence of 200 μM AL1 for 48 h at room temperature exhibited a 74% reduction in the ability to bind to human bladder cells compared with non-treated bacteria (*P* = 0.0003, *n* = 2 independent experiments, each carried out in triplicate) (Figure 4). Under the same conditions, bladder cell binding of UTI89 Δ*fim* was reduced by 84% relative to wild-type.

### AL1 causes a rapid decrease in surface-exposed type 1 pili

**Figure 5.**
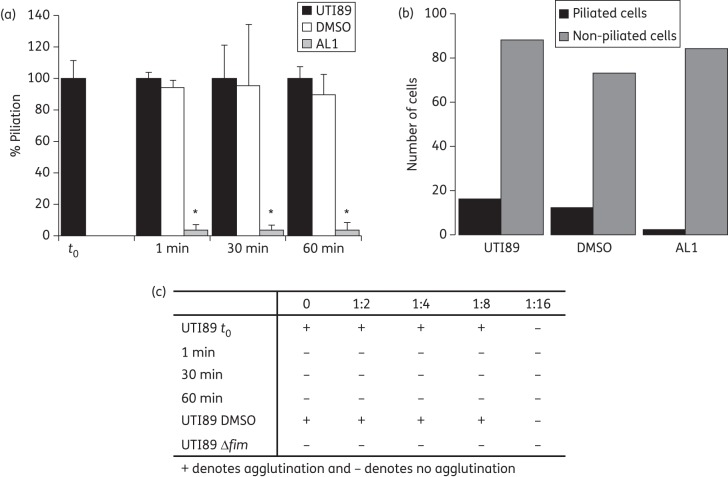
Timing of AL1 activity. (a) Degree of piliation as represented by FimH surface exposure on UTI89 grown on LB, measured by anti-FimH WCE on samples taken before (*t*_0_) and 1, 30 and 60 min following the addition of 200 μM AL1 + 1% DMSO or 1% DMSO alone, shown as mean ± SEM percentage relative to value for UTI89 at t_0_; *n* = 2 independent experiments with two samples each. *P = 0.0004, *P* = 0.0005 and *P* = 0.0004 for samples taken at 1, 30 and 60 min following the addition of 200 μM AL1, respectively; two-tailed unpaired Student's *t*-test against UTI89 *t*_0_. (b) Quantitative analyses of piliated and non-piliated UTI89 cells, assessed by electron microscopy, 1 min following the addition of 200 μM AL1 + 1% DMSO, 1% DMSO or UTI89 controls. (c) Agglutination titre of UTI89 at *t*_0_ and 1, 30 and 60 min following the addition of 200 μM AL1 + 1% DMSO or 1% DMSO alone. UTI89 Δ*fim* was used as a negative control. 0 denotes undiluted sample with a starting OD_600_ of 0.1. The image files for this assay are shown in Figure S7, available as Supplementary data at *JAC* Online.

**Figure 6.**
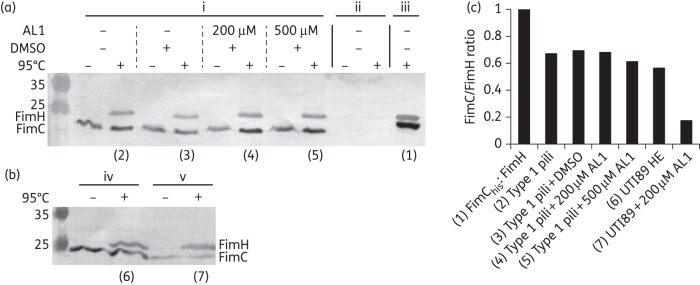
AL1 activity against mature type 1 pili. Western blot analysis of type 1 pili in the presence of AL1. (a) DMSO or AL1 treatment of type 1 pili (i) or negative control isolates (ii) obtained by MgCl_2_ precipitation of the supernatants of UTI89_LON and UTI89 Δ*fim*, respectively. (iii) Purified FimC:FimH. (b) MgCl_2_-precipitated supernatant of a UTI89_LON suspension that was heat shocked (iv) or treated with 200 µM AL1 for 1 min (v). Blots were probed with rabbit polyclonal antibody against FimC:FimH complex. (c) Bar graph presentation of the ratio of FimC to FimH for the different pilus isolates, normalized against the ratio of FimC_his_ to FimH for the purified, equimolar FimC_his_:FimH complex.

In each of the above experiments, AL1 was present throughout bacterial growth. To determine the timepoint at which AL1 becomes active, UTI89 was cultured in rich medium and exposed to 200 μM AL1 during the exponential growth phase. Cells were harvested at 1, 30 and 60 min post-treatment and assayed for FimH surface exposure using ELISA and type 1 pilus counts by electron microscopic visualization (Figure 5a and b). Based on a model in which AL1 exclusively acts on nascent pili, we predicted levels of surface-exposed FimH to drop by 50% per generation time (∼20 min). However, we found that surface-exposed FimH was reduced to background levels within 1 min after the addition of AL1 (Figure 5a). Indeed, electron microscopic inspection revealed that the AL1-treated cells had lost their type 1 pili within 1 min following exposure to the compound (Figure 5b). In AL1-treated cultures the level of piliated bacteria decreased to 2%, whilst DMSO-treated controls maintained near wild-type levels of pili (Figure 5b). The rapid loss of type 1 pili upon AL1 treatment was corroborated in a yeast agglutination assay. Whereas non-treated or DMSO-treated UTI89 showed an agglutination titre of 1 : 8, AL1-treated cultures showed a lack of yeast agglutination within 1 min post-treatment (Figures 5c and S7, available as Supplementary data at *JAC* Online). These observations indicate that pre-existing type 1 pili are lost from the bacterial surface upon addition of AL1 and suggest that the compound retains an inhibitory effect downstream of the FimH:FimG DSE reaction. To test whether AL1 disrupts pilus structure, type 1 pili isolated by heat extraction and MgCl_2_ precipitation^38^ were exposed to 200 and 500 µM compound. SDS–PAGE analysis showed that FimH remained pilus-bound and only migrated into the resolving gel when the samples were boiled in 1% SDS (Figure 6a). Thus, AL1 does not affect quaternary structure in preassembled pili. Chaperone–usher pili are tethered to the bacterial surface by anchoring to the outer membrane usher via the last incorporated chaperone:subunit complex.^19^ Possibly, AL1 binding to the basal subunit destabilizes the chaperone:subunit interaction, leading to the dissociation of the pili. In support of this hypothesis, we found that, in contrast to heat-extracted pili, type 1 pili found in the supernatant of an AL1-treated bacterial suspension contained pilus-bound FimH, but showed a drastically reduced stoichiometry of the FimC chaperone (Figure 6b and c).

### Modelling of the putative AL1 binding site

In order to gain insight into the interaction of AL1 with the FimH binding site, molecular docking was performed. A couple of low-energy docking poses were suggested by the GOLD docking. Their conformations, interactions in the binding pockets and GoldScore were very similar. The best docking pose of AL1, based on GoldScore, is shown in Figure S8 (available as Supplementary data at *JAC* Online). The structure of AL1 is quite flexible as it has many rotatable bonds. However, in the docked structure it always adopts a linear conformation, mimicking the N-terminus of FimG. This conformation has a good shape that is complementary to the binding pocket within FimH. There are two hydrogen bonds proposed by this pose: one hydrogen bond is formed between the sulphonamide oxygen atom and the backbone NH of Ile115 while another hydrogen bond is formed between the amide hydrogen and the hydroxyl group of Tyr99. The various binding poses differed in that AL1 shifts slightly along the pocket, maximizing the fit with the binding site. The two hydrogen bonds may not be very strong. In some poses they did not form at all or in others, a different hydrogen bond was formed between another hydrogen donor or acceptor within AL1 and a backbone NH or C = O from another residue. It is speculated that shape recognition and hydrophobic interactions between AL1 and the protein are more important. However, confirmation that AL1 indeed binds the FimH P5 area, and in what conformation it does so, will require future structural validation. Thus far, attempts at co-crystallizing AL1 bound to the chaperone:subunit complex have failed due to unfavourable crystal-packing contacts in the P5 area.

### The prophylactic and therapeutic advent of pilus attenuation

The compound identified in this study represents a novel inhibitor of type 1 pilus-mediated virulence in uropathogenic *E. coli*. The concept of anti-virulence therapies has gained significant momentum due to the increasing realization of the adverse effect of broad-acting bacteriostatic or bacteriolytic antibiotics.^4,14,45^ Furthermore, it is anticipated that targeted antibacterials could minimize selective pressure that perpetuates drug resistance.^14,46,47^ In UPEC-caused UTI, the rise in multiple antibiotic resistance, the high incidence of recurrent infections and the high burden of nosocomial UTIs, particularly in patients with urinary catheters, provide a strong impetus for the development of alternative therapeutic and prophylactic treatments. In recent years, potent receptor analogues that block binding of the FimH adhesin to its high-mannose receptors on the host have been developed.^48–50^ *In vivo* studies in a murine model of UPEC-caused UTI have since shown that type 1 pilus virulence attenuation is a viable therapeutic approach, reducing bacterial counts in the affected organs to numbers similar to those seen after treatment with commonly prescribed bacteriocidal or bacteriostatic antibiotics.^51,52^ This includes treatment of multidrug-resistant strains such as the recently emerged pandemic UPEC strain ST131.^9^ Moreover, the bacterial clearance through virulence attenuation proved synergistic with standard antibiotics.^51^ Besides type 1 pilus attenuation via receptor analogues, efforts have been made towards the development of pilus biogenesis inhibitors.^53^ A possible advantage of biogenesis inhibitors compared with receptor analogues is their use in the coating of urinary catheters in order to reduce the risk of catheter-associated UTIs (CAUTIs). In contrast, adsorption of receptor analogues on catheter walls risks attracting the attachment of type-1-positive bacteria to urinary catheters.

Finally, fimbrial adhesins form important virulence factors in a range of bacterial pathogens, most notably in human and animal enteropathogens such as *Shigella, Escherichia* and *Salmonella*.^54,55^ For many of these fimbrial adhesins, however, inhibition through receptor analogues promises to be problematic because of the presence of an avidity-based adhesion mechanism (e.g. polyadhesive binding with low affinity interactions—low micromolar to millimolar—for the individual adhesin–glycan pairs) and/or because of complex and shallow binding sites that show poor druggability.^4,14,45,56^ In such cases, direct elimination of the organelles through inhibition of their biosynthetic step, as demonstrated for the polymerization inhibitors identified in this study, could provide a promising alternative approach.

In summary, we have demonstrated that specific screening from *in silico* preselected compounds with predicted complementarity to the FimC:FimH donor strand acceptor groove yielded a specific inhibitor, AL1, that targeted the FimC:FimH DSE process. Accordingly, AL1 was shown to inhibit type 1 pilus biogenesis *in vivo* when added in low micromolar concentrations. Bacterial cells treated with AL1 instantaneously and completely lost type 1 pili. To the best of our knowledge, AL1 is the first small compound known to inhibit DSE. This study provides a basis for the further design and development of anti-virulence drugs targeting the DSE reaction mechanism, given its widespread conservation in the chaperone–usher assembly of a diverse array of cell surface-associated virulence determinants in many important bacterial pathogens.

## Funding

This work is supported by the Flanders Institute for Biotechnology (VIB) through grant PRJ9 and by the Fonds Wetenschappelijk Onderzoek-Vlaanderen (FWO) through Odysseus grant G.0902.09. D. S. and K. S. are supported by the Medical Research Council (MRC) grant 85602 to G. W.

## Transparency declarations

All authors: none to declare.

Data disclosed in this manuscript form the subject of patent application GB 1307233.5.

## Supplementary data

Additional methods, Table S1 and Figures S1 to S8 are available as Supplementary data at *JAC* Online (http://jac.oxfordjournals.org/).

Supplementary Data
