# Supplementary material for: Suppression of type 1 pilus assembly in uropathogenic Escherichia coli by chemical inhibition of subunit polymerization
Source: J Antimicrob Chemother. 2013 Dec 8;69(4):1017–26. doi: 10.1093/jac/dkt467 (PMC3956373; doi:10.1093/jac/dkt467)
Supplement: Supplementary Data [file supp_69_4_1017__index.html]

Suppression of type 1 pilus assembly in uropathogenic Escherichia coli by chemical inhibition of subunit polymerization — Suppression of type 1 pilus assembly in uropathogenic Escherichia coli by chemical inhibition of subunit polymerization — Supplementary Data 

# Suppression of type 1 pilus assembly in uropathogenic *Escherichia coli* by chemical inhibition of subunit polymerization

## Supplementary Data

Supplementary Data

**Files in this Data Supplement:**

- Supplementary Data - Doc file
