# Supplementary material for: Suppression of type 1 pilus assembly in uropathogenic Escherichia coli by chemical inhibition of subunit polymerization
Source: J Antimicrob Chemother. 2013 Dec 8;69(4):1017–26. doi: 10.1093/jac/dkt467 (PMC3956373; doi:10.1093/jac/dkt467)
Supplement: Supplementary Data [file supp_dkt467_dkt467supp.doc]

**Supplementary data**

**Synthetic protocols**

To confirm the identity of the biologically active compound in ASN-03798371 (Asinex corporation) we have synthesised N-(4-chlorophenyl)-2-((5-(4-(pyrrolidin-1-ylsulfonyl)phenyl)-1,3,4-oxadiazol-2-yl)thio)acetamide in-house (**6**; AL1*). All commercially available solvents and reagents were used without further treatment as received unless otherwise noted. NMR spectra were measured with a Bruker DRX 500 or 600 MHz spectrometer; chemical shifts are expressed in ppm relative to TMS as an internal standard and coupling constants (J) in Hz. Mass spectra were obtained using a Waters ZQ2000 single quadrupole mass spectrometer with electrospray ionisation (ESI). High resolution mass spectra were acquired on a Waters LCT time of flight mass spectrometer with electrospray ionisation (ESI) or chemical ionization (CI). Analysis of intermediates by reverse-phase LCMS was carried out on an analytical C18 column (Phenomenex Gemini, 50 x 3.0 mm, 5 μm) and an AB gradient of 5–95 % for B at a flow rate of 1 mL/minute, where eluent A was 0.1 % formic acid/water and eluent B was 0.1 % formic acid/acetonitrile. Routine analytical thin layer chromatography was performed on pre-coated plates (60F254, Machery-Nagel).

**General synthetic scheme**

*Reagents and conditions*: a) pyrrolidine, 10 % NaOH, room temperature, 16 hrs, 56% yield; b) i) *t*-butyl carbazate, EDC.HCl, CH_2_Cl_2_, room temperature, 16 hrs, 71% yield; c) TFA, 0 ^o^C, 72% yield; d) KOH, EtOH, CS_2_, reflux,18 hrs, 76% yield; e) BrCH_2_CO_2_H, NMM, EtOH, room temperature, 72 hrs, 54% yield; f) *p*-chloroaniline, EDC.HCl, CH_2_Cl_2_, rt, 1 hr, 78% yield

**4-(Pyrrolidin-1-ylsulfonyl)benzoic acid (1)**

To a stirred solution of pyrrolidine (0.75 mL, 9.00 mmol) in NaOH (10.0 mL, aq. 10% soln.) was added 4-(chlorosulfonyl)benzoic acid (1.00g, 4.50 mmol). The reaction was stirred at room temperature for 16 hours, then acidified with conc. HCl. The resulting precipitate was filtered, washed with more HCl (1M), and dried on frit to give **1** as a white solid (0.64 g, 56%). Compound **1** was used without further purification.

^1^H NMR (500 MHz, (CD_3_)_2_SO)) δ; 13.50 (s, 1H), 8.15 (d, *J* = 8.6 Hz, 2H), 7.92 (d, *J* = 8.6 Hz, 2H), 3.16 (t, J = 6.74 Hz, 4H), 1.65 (m, 4H); ^13^C NMR (150 MHz, (CD_3_)_2_SO)) δ; 166.3 (*C*OOH), 139.9, 134.6, 130.3, 127.6, 47.9 (*C*H_2_), 24.78 (*C*H_2_); HRMS (*m/z*): [M-H]^-^ calculated for C_11_H_12_NO_4_S, 254.0487; found, 254.0486.

***Tert*-butyl 2-(4-(pyrrolidin-1-ylsulfonyl)benzoyl)hydrazinecarboxylate (2)**

To a stirred solution of **1** (3.84 g, 15.6 mmol) in CH_2_Cl_2_ (15 mL) was added *t*-butyl carbazate (2.14 g, 16.2 mmol) and EDC.HCl (3.11 g, 16.2 mmol). The reaction was stirred for 16 hours at room temperature. The reaction mixture was then washed with NaHCO_3_ (3 x 15 mL, satd. soln.), NaCl (15 mL, satd. soln.) extracted with CH_2_Cl_2_ (50 mL) and dried over MgSO_4_. The product was purified by flash silica chromatography (EtOAc:cyclohexane, 2:8) to give **2** as a white crystalline solid (3.60 g, 71% yield).

^1^H NMR (500 MHz, (CDCl_3_)) δ; 9.18 (s, 1H), 7.92 (d, *J* = 8.3 Hz, 2H), 7.78 (d, *J* = 8.3 Hz, 2H), 3.20 (t, J = 6.7 Hz, 4H), 1.73 (m, NCH_2_C*H*_2_, 4H); 1.45 (s, 9H); ^13^C NMR (150 MHz, (CDCl_3_)) δ; 171.5 (*C*=O), 156.2 (*C*=O), 140.2, 135.5, 128.3, 127.7, 82.5 (*C*-(CH_3_)_3_), 48.1 (*C*H_2_), 28.3 ((*C*H_3_)_3_), 25.3 (*C*H_2_); HRMS (*m/z*): [MH]^+^ calculated for C_16_H_23_N_3_O_5_S, 370.1431; found, 370.1417.

**4-(pyrrolidin-1-ylsulfonyl)benzohydrazide (3)**

**2** (0.93g, 2.50 mmol) was cooled to 0^o^C and TFA (5 mL) was added dropwise. The mixture was stirred and allowed to warm to room temperature over 1 hour. After this time NaHCO_3_ (satd. soln.) was added until effervescence ceased. Product was then extracted with CH_2_Cl_2_ (50 mL) and washed with further NaHCO_3_ (4 X 25 mL, satd. soln.). Solvent was then removed under reduced pressure to give **3** as a white solid (0.48 g, 72% yield). Compound **3** was used without further purification.

**5-(4-(pyrrolidin-1-ylsulfonyl)phenyl)-1,3,4-oxadiazole-2-thiol (4)**

To a stirred solution of **3** (0.51 g, 1.90 mmol) in ethanol (20 mL) was added KOH (0.11 g, 2.00 mmol) and the mixture was stirred at room temperature for 5 minutes. Carbon disulfide (0.18 mL, 3.00 mmol) was then slowly added to the mixture while the reaction was heated to reflux. The reaction was then stirred under reflux for 18 hours. After this time the reaction was allowed to cool to rt and solvent was removed under reduced pressure. The residue was then dissolved in water (25 mL) and HCl (1 M) added until a white precipitate formed. The precipitate was filtered and dried on frit to give **4** as a white solid (0.45g, 76%). Compound **4** was used without further purification.

^1^H NMR (600 MHz, (CD_3_CN)) δ; 12.15 (s, 1H), 8.08 (d, *J* = 8.1 Hz, 2H), 7.95 (d, *J* = 8.1 Hz, 2H), 3.22 (t, *J* = 6.3 Hz, 4H), 1.71 (t, *J* = 6.3 Hz, 4H); ^13^C NMR (150 MHz, (CDCl_3_) δ; 179.4 (O*C*SH), 160.7 (O*C*=N), 140.9, 129.2, 127.9, 127.6, 49.0 (*C*H_2_), 25.9 (*C*H_2_); HRMS (*m/z*): [M]^+^ calculated for C_12_H_13_N_3_O_3_S_2_, 311.0392; found, 311.0383.

**2-((5-(4-(pyrrolidin-1-ylsulfonyl)phenyl)-1,3,4-oxadiazol-2-yl)thio)acetic acid (5)**

To a stirred solution of **4** (0.05 g, 0.15 mmol) in ethanol (5 mL) was added NMM (0.02 mL, 0.20 mmol) and bromoacetic acid (0.03 g, 0.17 mmol). Reaction was stirred at room temperature for 72 hours. After this time solvent was removed under reduced pressure, and the residue redissolved in CH_2_Cl_2_ (25 mL) and washed with HCl (1 M, 20 mL). The organic layer was separated, dried over magnesium sulfate and filtered. Solvent was removed under reduced pressure to give **5** as a white sold (0.03 g, 54%). Compound **5** was used without further purification.

**N-(4-chlorophenyl)-2-((5-(4-(pyrrolidin-1-ylsulfonyl)phenyl)-1,3,4-oxadiazol-2-yl)thio)acetamide (6)**

To a stirred solution of **5** (0.03 g, 0.08 mmol) in CH_2_Cl_2_ (5 mL) was added EDC.HCl (0.020 g, 0.10 mmol) and *p*-chloroaniline (0.01 g, 0.10 mmol). The reaction was stirred at room temperature for 1 hour before being washed with HCl (1 M) and extracted with further CH_2_Cl_2_ (50 mL). The product was purified by flash silica chromatography (EtOAc:cyclohexane, 2:8) to give **6** as a white solid (0.03 g, 78%).

^1^H NMR (600 MHz, (CD_3_)_2_SO)) δ; 10.61 (s, 1H), 8.18 (d, *J* = 8.3 Hz, 2H), 7.99 (d, *J* = 8.3 Hz, 2H), 7.62 (d, *J* = 8.8 Hz, 2H), 7.39 (d, *J* = 8.8 Hz, 2H), 4.38 (s, 2H), 3.18 (t, *J* = 6.6 Hz, 4H), 1.65 (t, *J* = 6.6 Hz, 4H); ^13^C NMR (150 MHz, (CD_3_)_2_SO)) δ: 165.1 (*C*ONH), 164.4 (*C*S), 164.2 (*C*ON), 138.9 (*C*SO_2_), 137.6 (*C*-NH), 128.9, 128.3, 127.3 (signals overlapping), 126.7 (*C*Cl), 120.7, 47.94 (N*C*H_2_), 36.83 (S*C*H_2_), 24.80 (*C*H_2_CH_2_N); HRMS (*m/z*): [MH]^+^ calculated for C_20_H_20_ClN_4_O_4_S_2_, 479.0609; found, 479.0610.

**Table S1.** List of strains, plasmids and primers used in this study

| **Strain, plasmid or primer** | **Relevant description or sequence (5'–3')** | **Reference(s) or source** |
| --- | --- | --- |
|  |  |  |
| **Strains** |  |  |
| UTI89 | *E. coli* strain UTI89, a human cystitis isolate | 1 |
| UTI89Δfim | UTI89Δ*fimBEAICDFGH* | 2 |
| UTI89_LON | *E. coli* strain UTI89, a mutant constitutively expresses type 1 pilus | 3 |
| BL21(DE3) | *F^-^ ompT gal dcm lon hsdSB*(*rB^-^ mB^-^*) (*lacIq lacUV5-T7 ind1 sam7 nin5 λcIts857*) | 4 |
|  |  |  |
| **Plasmids** |  |  |
| pETS1000 | *fimC* with six C-terminal His-tag codons in pMON6235Δcat, Spec^r^ | 5 |
| pKVWc1 | pDONRP4-P1R-*fimC* with six C-terminal His-tag codons, Kan^r^ | This study |
| pENT53 | pDONR221-*fimH*, Kan^r^ | This study |
| pENT105 | pDONRP2R-P3-*gfp+*, Kan^r^ | A gift from Henri De Greve |
| pDEST22a |  | This study |
| pKVWe1 | pDEST22a-*fimC* with six C-terminal His-tag codons *fimH gfupv*, Amp^r^ | This study |
|  |  |  |
| **Primers** |  |  |
| FimCf | GGGGACAACTTTGTATAGAAAAGTTGTCGAAGGAGATAGAACCATGAGTAATAAAAACGTCAATGTAAGG | This study |
| FimCr | GGGGACTGCTTTTTTGTACAAACTTGCTTAGTGATGGTGATGGTGATGTTCCATTACGCCCGTCATTTTGG | This study |
| FimH3 | GGGGACAAGTTTGTACAAAAAAGCAGGCTTCAGAAGGAGATATATCATGAAACGAGTTATTACCCTGTTTGCTGT | This study |
| FimH7 | GGGGACCACTTTGTACAAGAAAGCTGGGTTTTATTGATAAACAAAAGTCACGCCAATA | This study |
| gfp7 | GGGGACAGCTTTCTTGTACAAAGTGGTCGAAGGAGATAGAACCATGAGTAAAGGAGAAGAACTTTTCAC | This study |
| gfp8 | GGGGACAACTTTGTATAATAAAGTTGCCTTATTTGTAGAGCTCATCCATGCCATG | This study |

**References**

1. Chen, S.L. et al. Identification of genes subject to positive selection in uropathogenic strains of Escherichia coli: a comparative genomics approach. *Proc Natl Acad Sci U S A* **103**, 5977-82 (2006).

2. Derous, V., Deboeck, F., Hernalsteens, J.P. & De Greve, H. Reproducible gene targeting in recalcitrant Escherichia coli isolates. *BMC Res Notes* **4**, 213 (2011).

3. Kostakioti, M. et al. Distinguishing the contribution of type 1 pili from that of other QseB-misregulated factors when QseC is absent during urinary tract infection. *Infect Immun* **80**, 2826-34 (2012).

4. Studier, F.W. & Moffatt, B.A. Use of bacteriophage T7 RNA polymerase to direct selective high-level expression of cloned genes. *J Mol Biol* **189**, 113-30 (1986).

5. Saulino, E.T., Thanassi, D.G., Pinkner, J.S. & Hultgren, S.J. Ramifications of kinetic partitioning on usher-mediated pilus biogenesis. *EMBO J* **17**, 2177-85 (1998).

**
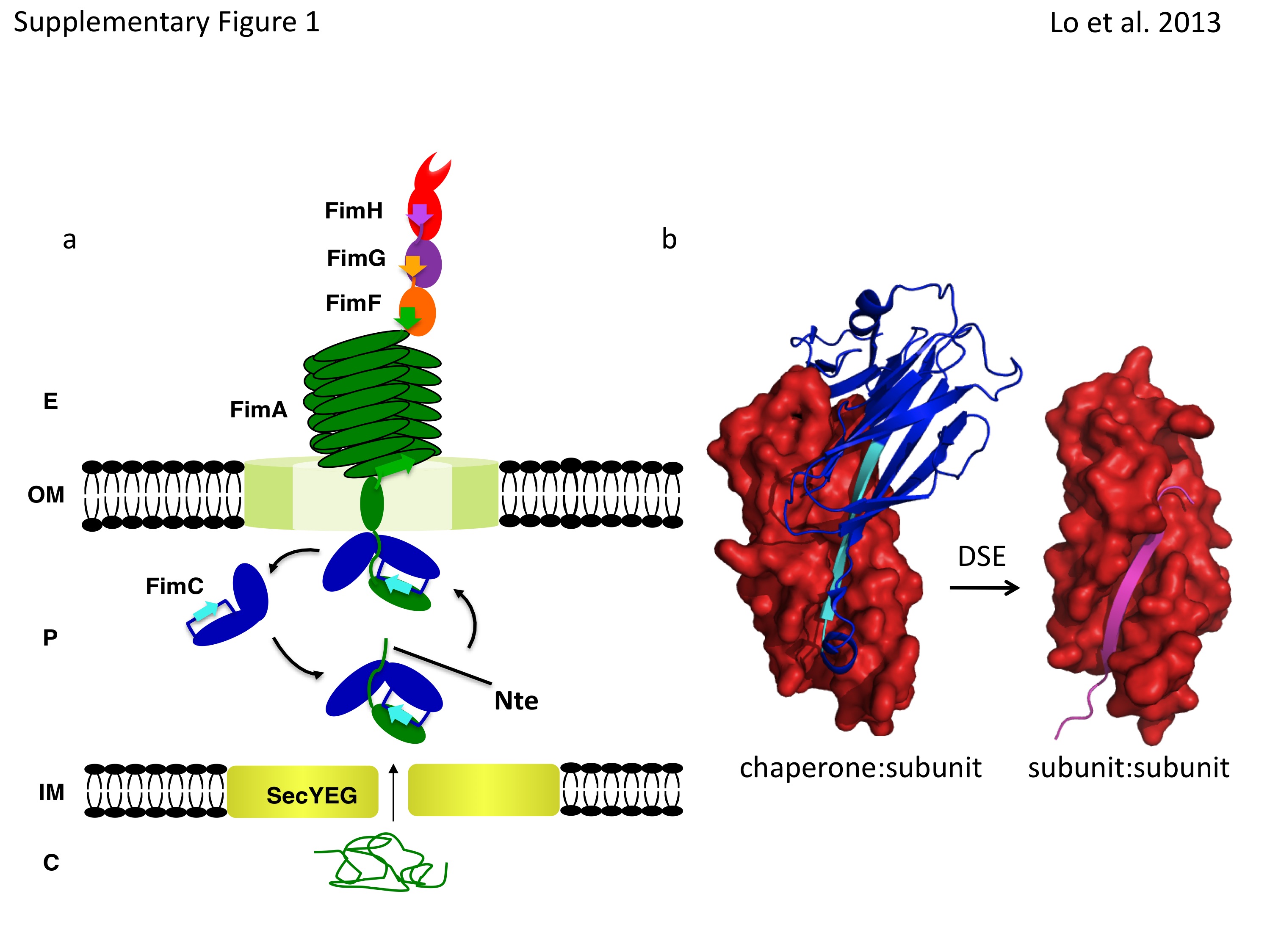
**

**Figure S1.** (a) Schematic presentation of type 1 pilus CU pathway and its subunits undergoing donor-strand-exchange (DSE). CU pilus subunit translocate to the periplasmic space through the SEC translocon, where they form a binary complex with a periplasmic chaperone (FimC). Chaperone:subunit complexes are recruited to the usher (FimD), the pilus assembly platform in the outer membrane. At the usher, subunits undergo dono-strand-exchnage and are build into the growing fibre. During DSE, the N-terminal extension on an incoming subunit displaces the chaperone bound to the subunit at the base of the fibre. (b) X-ray structures the FimH pilin domain (residues 158-279; shown as red molecular surface) in complex with the FimC chaperone (left; taken from PDB:1ZE3; FimC and the FimC G1 strand are shown in blue and cyan ribbon representation, respectively). FimH pilin domain (residues 158-279; shown as red molecular surface) in complex with the FimG Nte (right; taken from PDB:3JWN; FimG Nte is shown in pink ribbon representation).

**
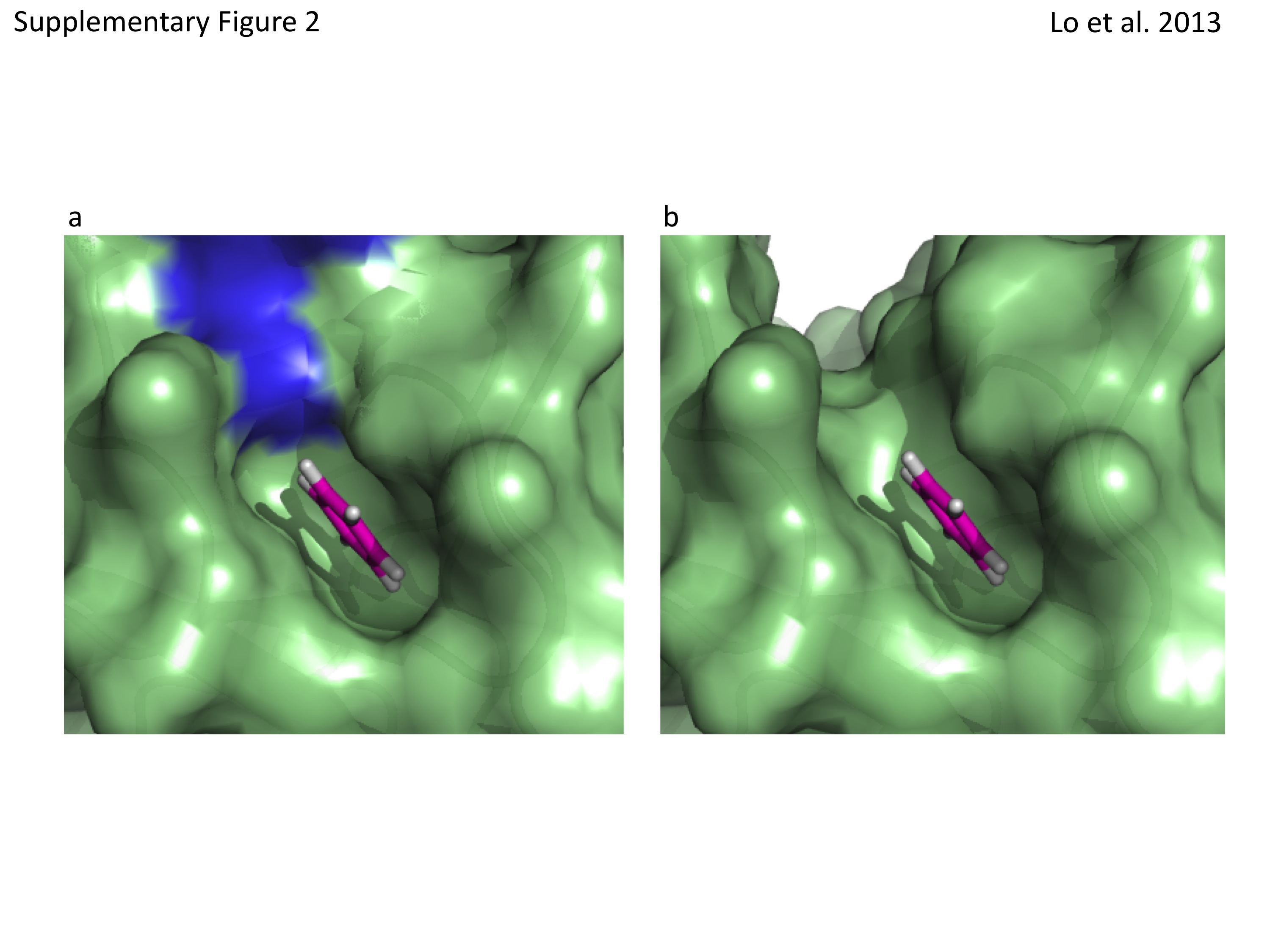
**

**Figure S2.** *In silico* screening of small compounds. Two strategies have been employed. (a) First, the docking screening of small compounds against the P5 pocket of FimC-FimH complex. (b) Second, FimC was removed from complex, a hydrophobic anchor was placed at the P5 pocket and docking of the full binding groove was carried out. The anchor was used to force binding near the P5 pocket to stimulate the unzipping of FimC away from the pocket.

**
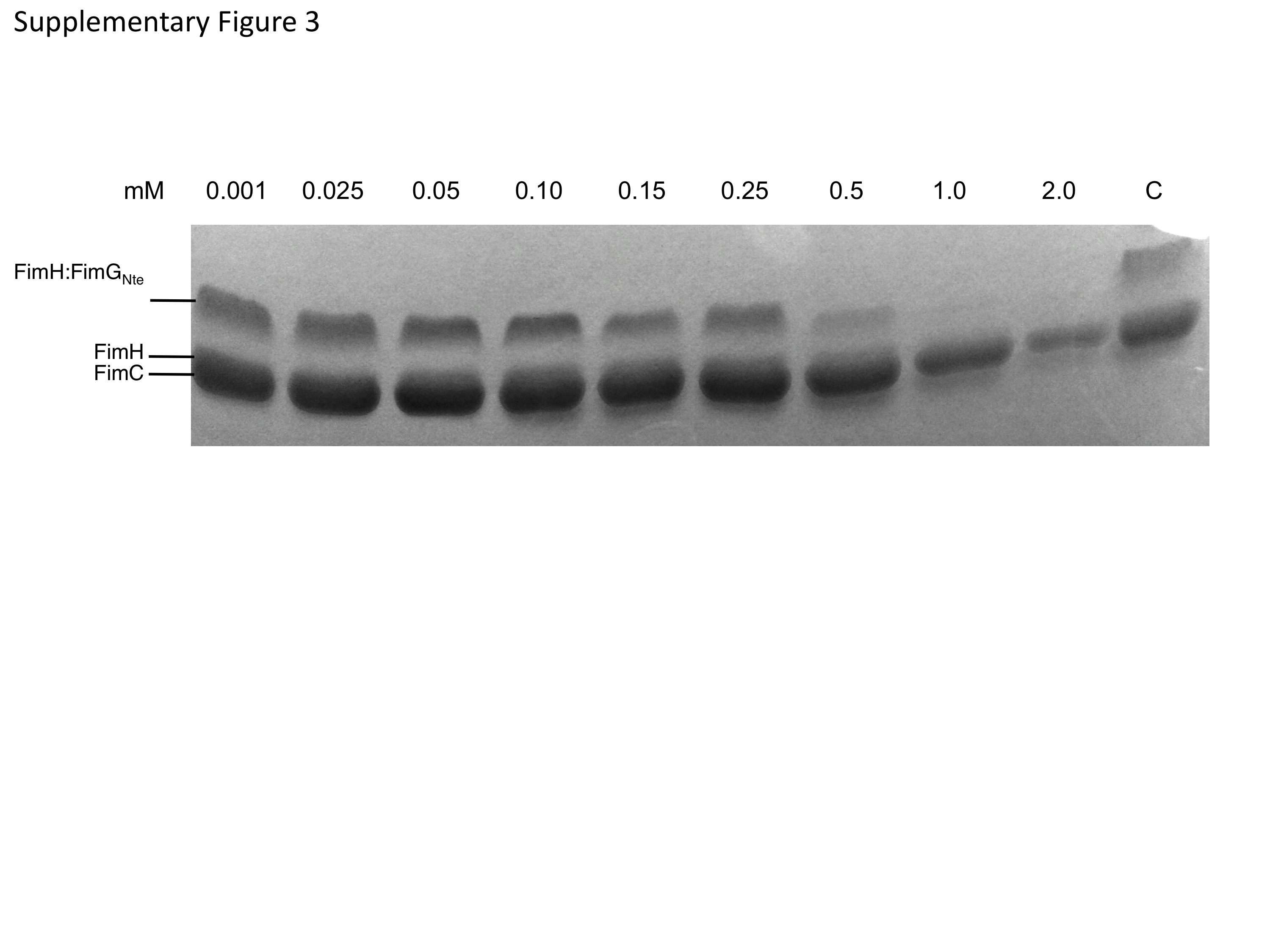
**

**Figure S3.** SDS-PAGE gel analysis of the DSE reactions of Fim:FimH and FimG Nte in the presence of AL1 at varying concentrations. The inhibitory activities AL1 were monitored by the formation of SDS-stable complex between FimH and the FimG Nte. C denotes non-treated control in the presence of DMSO (20% v/v).

**
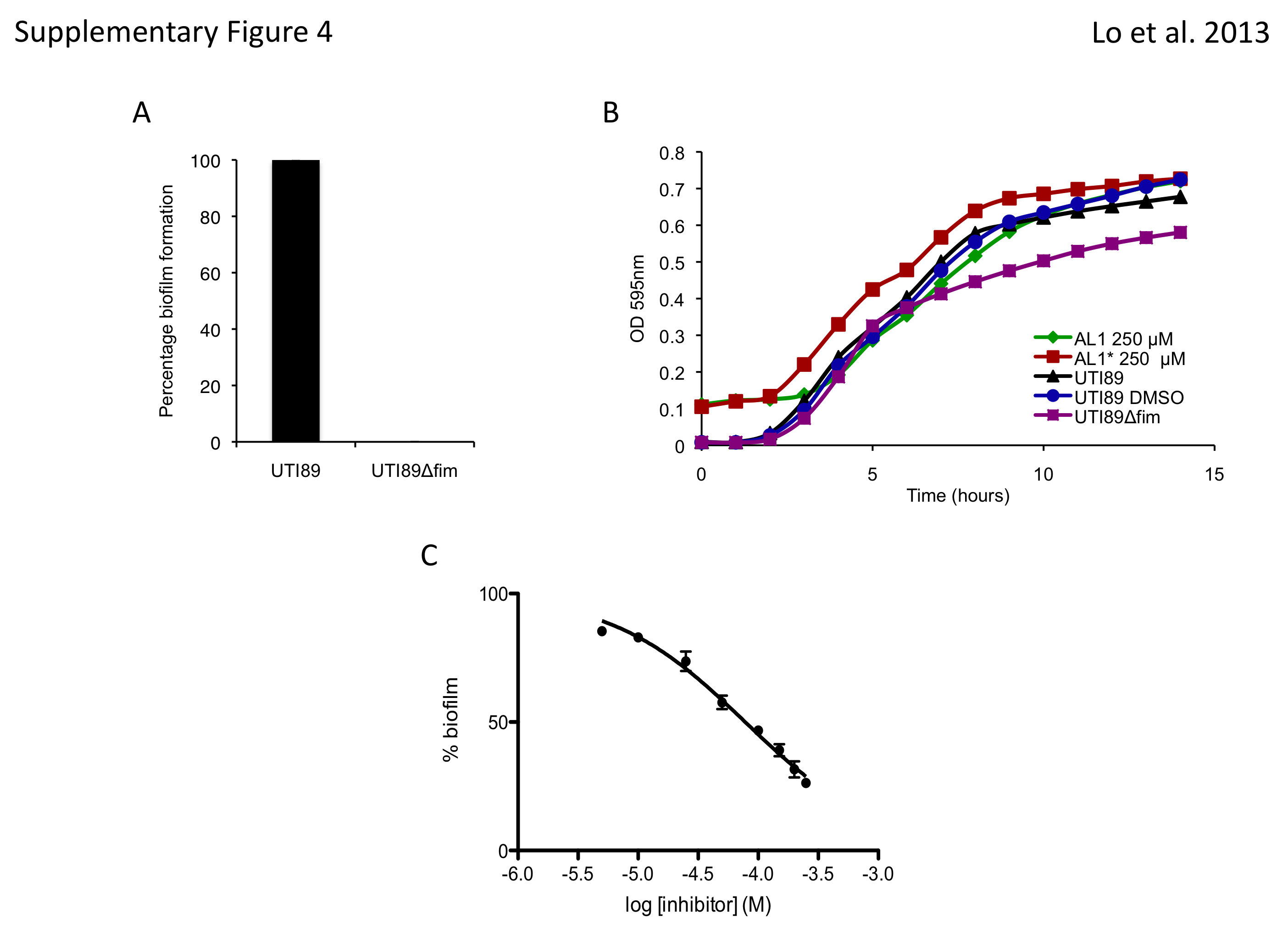
**

**Figure S4.** (a) Percent biofilm formation of *E. coli* strain UTI89 and UTI89Δ*fim* under the same static growth condition as AL1 treated cells in 96-well microtitre plate for 48 h at room temperature. (b) Growth curves of *E. coli* strain UTI89 and UTI89Δ*fim* grown in LB at 37 °C, monitored at OD595nm. UTI89 grown in LB medium containing 250 μM AL1, 250 μM AL1*, 1.25 % DMSO or 1.25 % DMSO exhibited comparable growth kinetics as UTI89 grown in LB alone. (c) Inhibition of UTI89_LON biofilm formation in the presence of AL1 at varying concentrations, plotted as percent biofilm formation relative to non-treated UTI89_LON. Data are presented as sample mean ± s.e.m., *n* = 3. Curves show non-linear regression fits with Hill slope of -1.4.

**
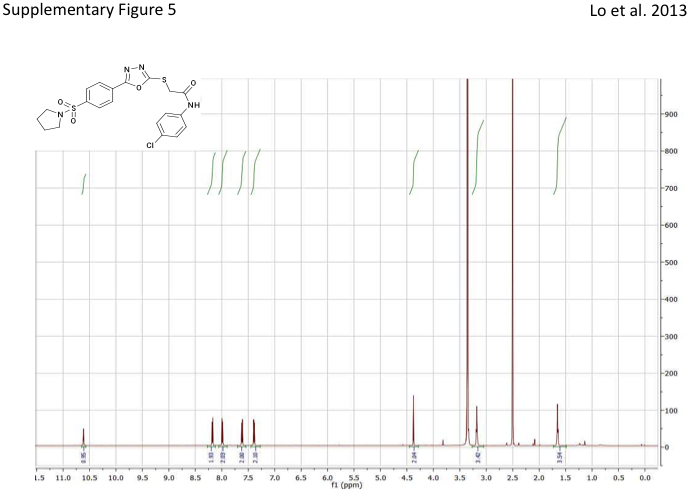
**

**Figure S5.** ^1^H NMR Spectrum of in-house synthesised AL1 (**6**).


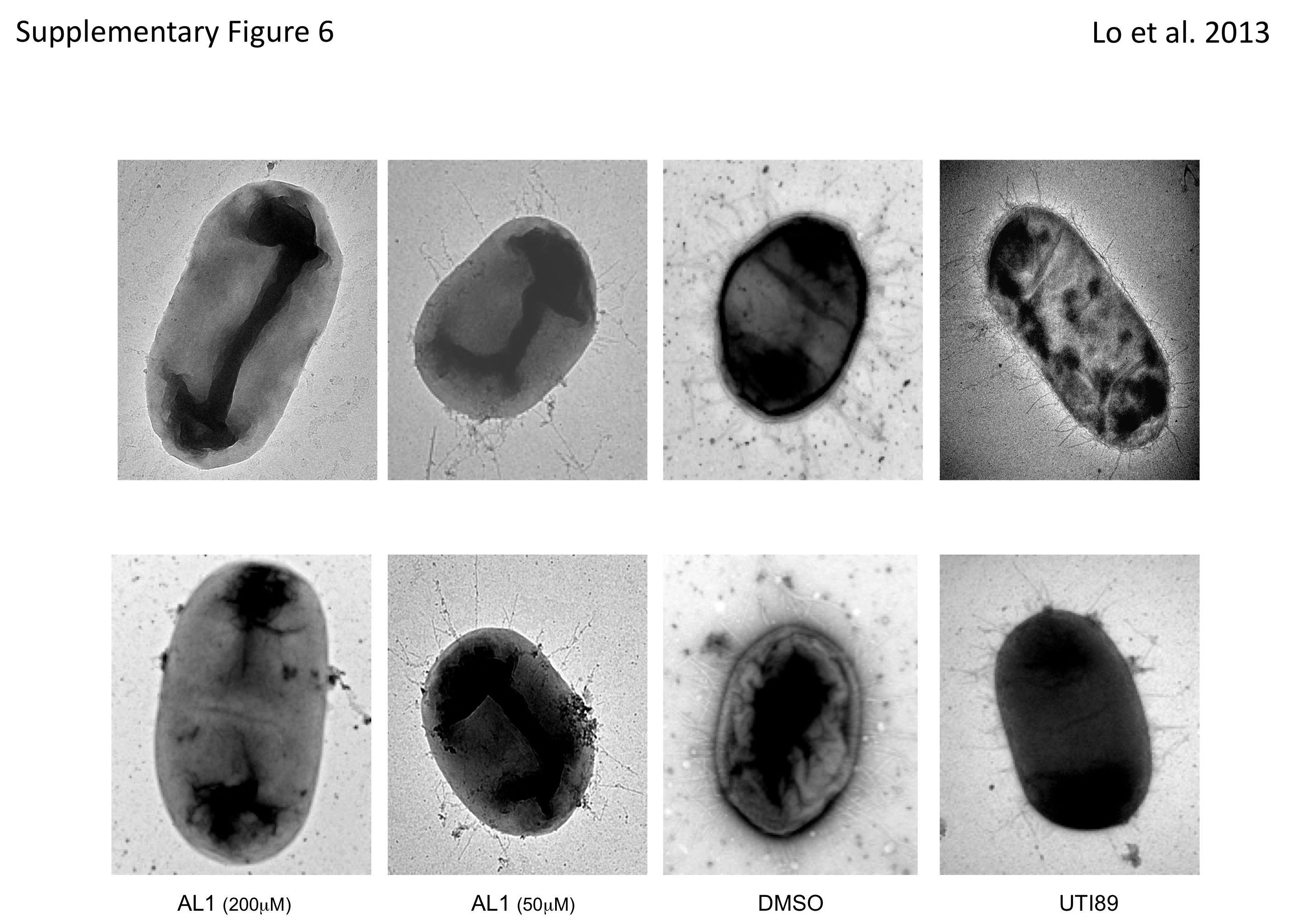
**Figure S6.** Representative electron micrographs of UTI89 grown in the presence of 50 µM or 200 µM AL1 + 1% DMSO, 1% DMSO, or non-supplemented LB, respectively.


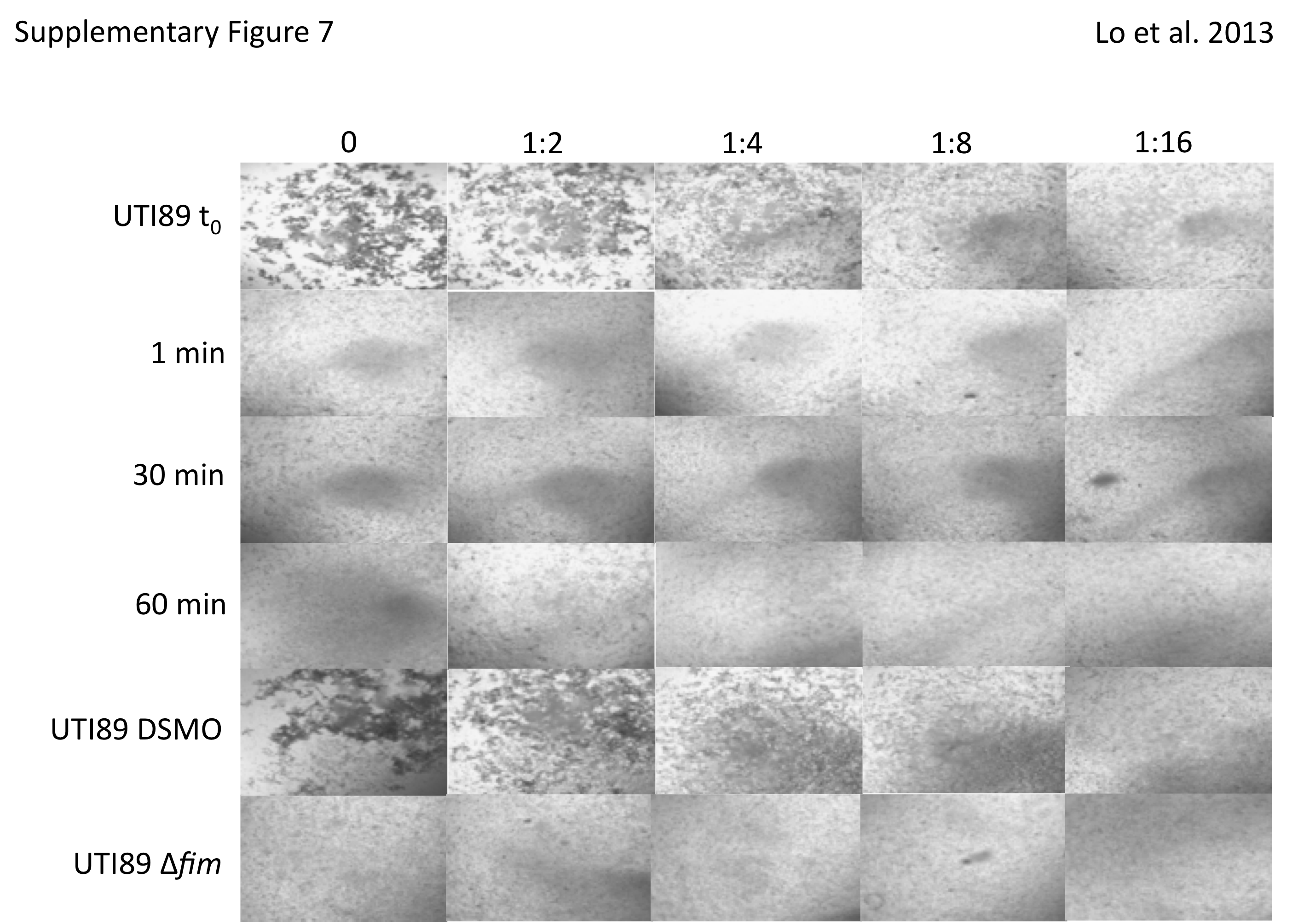
**Figure S7.** Images of the agglutination titre of UTI89 at t_0_ and 1, 30 and 60 minutes following the addition of 200 μM AL1 + 1% DMSO or 1% DMSO alone. UTI89Δ*fim* was used as a negative control. 0 denotes undiluted bacterial suspension with a starting OD_600nm_ of 0.1.

**
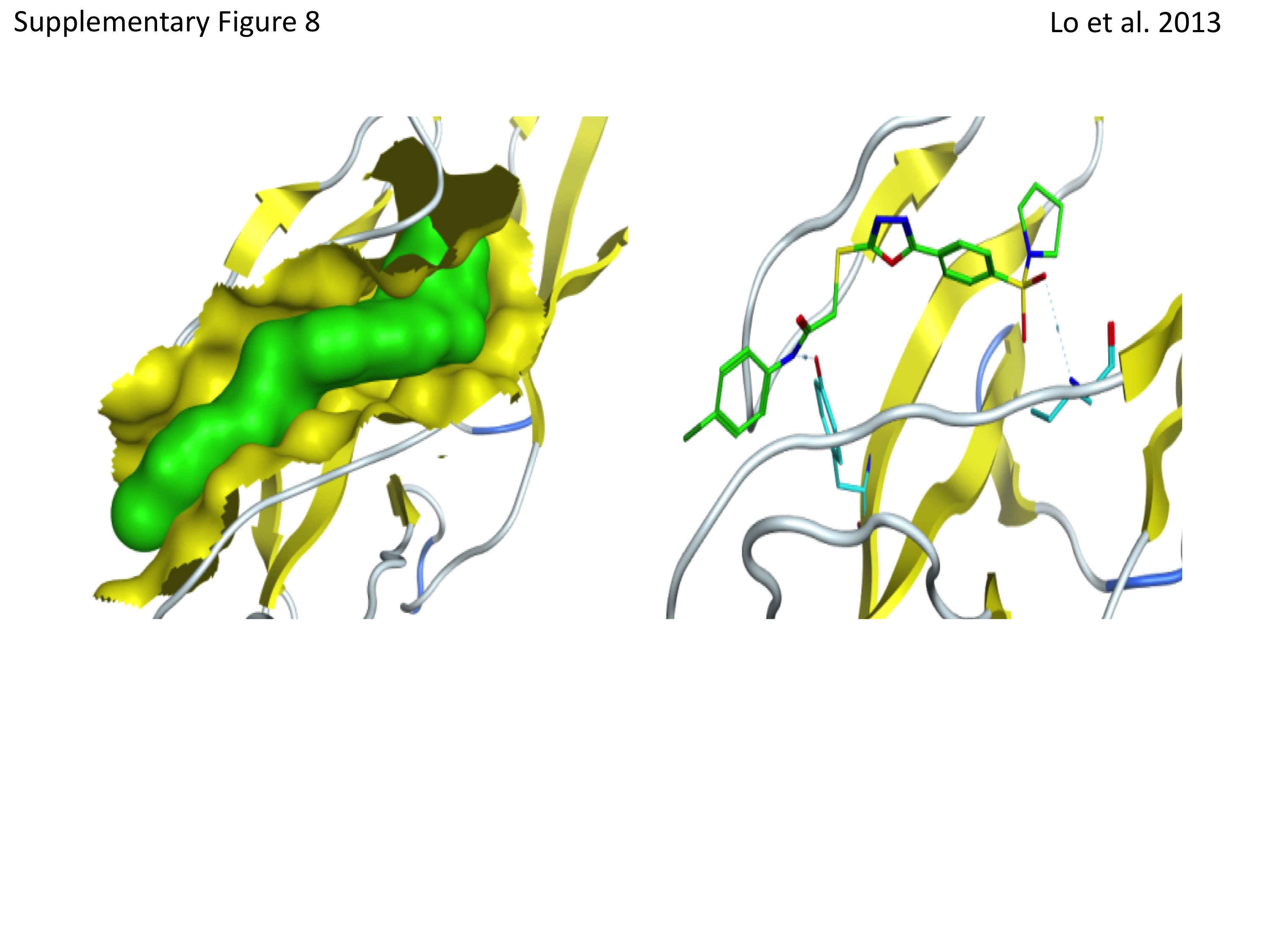
Figure S8.** Modeled interaction between AL1 and its putative binding site on FimH The surface of the binding pocket of FimH is shown in yellow, while the molecular surface of AL1 is shown in green, showing the complementarity of their shapes. Two hydrogen bonds were predicted in this docking pose: one with the hydroxyl group in Tyr99, the other with the backbone NH of Ile115 (hydrogen bonds shown as blue dashes). Carbon atoms in AL1 are in green, while those in FimH are in cyan.
